# The mystery of the cerebellum: clues from experimental and clinical observations

**DOI:** 10.1186/s40673-018-0087-9

**Published:** 2018-03-29

**Authors:** Charlotte Lawrenson, Martin Bares, Anita Kamondi, Andrea Kovács, Bridget Lumb, Richard Apps, Pavel Filip, Mario Manto

**Affiliations:** 1 School of Physiology, Pharmacology and Neuroscience, Biomedical Sciences Building, University of Bristol, Tankard’s Close, University Walk, Bristol, BS8 1TD UK; 2 First Department of Neurology, Faculty of Medicine, Masaryk University and St. Anne’s Teaching Hospital, Brno, Czech Republic; 3 Department of Neurology, School of Medicine, University of Minnesota, Minneapolis, USA; 4 Department of Neurology, National Institute of Clinical Neurosciences, Amerikai út 57, Budapest, 1145 Hungary; 5 Department of Neurology, Semmelweis University, Üllői út 26, Budapest, 1083 Hungary; 6 János Szentágothai Doctoral School of Neurosciences, Semmelweis University, Üllői út 26, Budapest, 1083 Hungary; 7 FNRS ULB-Erasme, 808 Route de Lennik, 1070 Bruxelles, Belgium; 8 Service des Neurosciences, UMons, 7000 Mons, Belgium; 9 Department of Neurology, Centre Hospitalier Universitaire (CHU) de Charleroi, 6000 Charleroi, Belgium; 10 Laboratoire de Médecine Expérimentale, Site Vésale, ULB Unité 222, 6110 Montigny-le-Tilleul, Belgium

**Keywords:** Cerebellum, Anatomy, History, Fear, Cognition, Motor, Timing, Tremor

## Abstract

The cerebellum has a striking homogeneous cytoarchitecture and participates in both motor and non-motor domains. Indeed, a wealth of evidence from neuroanatomical, electrophysiological, neuroimaging and clinical studies has substantially modified our traditional view on the cerebellum as a sole calibrator of sensorimotor functions. Despite the major advances of the last four decades of cerebellar research, outstanding questions remain regarding the mechanisms and functions of the cerebellar circuitry. We discuss major clues from both experimental and clinical studies, with a focus on rodent models in fear behaviour, on the role of the cerebellum in motor control, on cerebellar contributions to timing and our appraisal of the pathogenesis of cerebellar tremor. The cerebellum occupies a central position to optimize behaviour, motor control, timing procedures and to prevent body oscillations. More than ever, the cerebellum is now considered as a major actor on the scene of disorders affecting the CNS, extending from motor disorders to cognitive and affective disorders. However, the respective roles of the mossy fibres, the climbing fibres, cerebellar cortex and cerebellar nuclei remains unknown or partially known at best in most cases. Research is now moving towards a better definition of the roles of cerebellar modules and microzones. This will impact on the management of cerebellar disorders.

## Background

There is growing recognition of the critical importance of the cerebellum in motor control and cognitive operations. This paper aims to provide a concise overview of various approaches for a better understanding of the links between cerebellar neurobiology and clinical neurology. The selected four directions in cerebellar research illustrate the multiple pathways to elucidate cerebellar functions.

## The cerebellum and fear behaviour: clues from rodent studies

The cerebellum is the largest sensorimotor structure in the brain having extensive connections with the brainstem and spinal cord. Less widely discussed are its various projections to and from limbic regions including the amygdala, hypothalamus, prefrontal cortex and periaqueductal grey (PAG) [1, 2]. These structures play a role in fear behaviours such as fight, flight and freezing. To date there are few studies which have investigated cerebellar contributions to these behaviours, although such a role is suggested in human studies showing changes in the regulation of emotional control after a cerebellar lesion [3, 4]. The cerebellum is a highly compartmentalised structure, comprising multiple olivo-cortico-nuclear modules [5], and our working hypothesis is that individual modules (or perhaps multiple modules working together) are related to the integrated pattern of motor, autonomic and cognitive responses associated with defensive behaviours e.g. freezing behaviour elicited by a threat and the accompanying changes in cardiorespiratory activity and affective state.

### Cerebellar contributions to motor output in fear behaviour

In rodents, fear is commonly measured by the amount of freezing behaviour that occurs in the presence of a natural threat (e.g. a predator); freezing is characterised by a fixed, tense (immobile) posture. Stimulation of different cerebellar regions (vermal lobules IV, V, superior cerebellar peduncle, interpositus nucleus) in the rat have been found to elicit freezing-like responses [6], although the freezing was not quantified or specifically associated with fear. An initial freezing period was observed during early stimulation trials, but in later trials the animals would begin to sniff and move around. This suggests a process of adaptation to the stimulation and that the cerebellum may modulate other pathways responsible for driving the freezing behaviour.

Early reports also showed that lesions of the cerebellar vermis (probably corresponding to the A module) reduced freezing behaviour in rats in response to the presence of cat odour [7]. Similarly, and more recently, localised lesions confined to the lateral vermal lobule VIII, corresponding to the A2 module, cause a reduction in freezing behaviour during both innate and conditioned fear tasks [8].

More specifically, cerebellar vermal lobule VIII in the rat has been shown to receive powerful physiological connections from the ventrolateral PAG (vlPAG) via the inferior olive [8] and anatomically, the vlPAG receives reciprocal projections via the fastigial nuclei (a major output nucleus of the cerebellar vermis [9]). A similar pathway between the PAG and cerebellum has also been shown in humans using diffusion tensor MRI tractography [10].

It is widely thought that the PAG drives the appropriate behavioural response to a threat. For example, lesions of the vlPAG result in freezing deficits in rats [11, 12], and spike-triggered EMG averaging has found that single unit activity in the PAG occurs approximately 10 ms prior to increases in neck muscle activity during freezing, consistent with the PAG driving motor outflow [13]. However, this correlation was evident only for the initial phase of a freezing response, suggesting that other structures, including perhaps the cerebellum, may be recruited to sustain freezing. Thus, a range of anatomical, electrophysiological and behavioural studies point to the cerebellum being involved in the motor responses, particularly freezing, associated with emotional behaviour.

Nonetheless it is important to note that such findings are confined mainly to observations in rats. In the cat, electrical stimulation of the fastigial nuclei does not elicit freezing behaviour but instead causes hypertension and predatory attack [14]. This may reflect important species-specific differences in behavioural response to a threat [15].

### The cerebellum and autonomic responses in fear

The cerebellum is also strongly associated with changes in autonomic output e.g. cerebellar stimulation in awake and decerebrate animals results in changes in blood pressure and respiration [16–19]. Lesions of the cerebellar vermis in rats before or after fear conditioning can also impair the changes in heart rate normally observed when a conditioned stimulus is paired with an unconditioned stimulus [20]. Furthermore, humans with medial cerebellar lesions show an impairment in heart rate changes associated with auditory cued fear conditioning compared with healthy subjects [21]. The cerebellum therefore may contribute to both the motor (freezing) and autonomic aspects of a defensive response to fear.

### The cerebellum and affective fear

It remains unclear whether the cerebellum also plays a role in the affective state associated with defensive behaviours. The evidence outlined above is consistent with the cerebellum driving the appropriate motor and autonomic responses to a threatening stimulus but not necessarily being involved in the emotional aspects of the behaviour. In human studies, using fMRI, it has been shown that the presentation of positive and negative emotional faces results in haemodynamic changes in different lobules in the cerebellum [21, 22], suggesting a cerebellar role in emotion. However, invasive studies using animal models show a causal role of the cerebellum in affect are lacking. Nonetheless, Sacchetti and colleagues have provided direct evidence that the cerebellum in rodents is involved in the consolidation of fear memories [23, 24]. For example, reversible inactivation of the cerebellar vermis (lobules IV, V, VI) and interpositus nuclei using tetrodotoxin (TTX) at different time points following a conditioned fear paradigm showed that early inactivation (0-192 h), but not late inactivation (384 h) following conditioning, prevented freezing behaviour during retrieval. Their findings show that manipulations of the cerebellum manipulate the memory trace and not the expression of the motor response.

### How mossy fibres and climbing fibres contribute to fear behaviours

Overall it appears that the cerebellum is involved in multiple processes associated with an orchestrated response to fear: i) motor output in relation to freezing behaviour; ii) autonomic output in relation to associated changes in cardiorespiratory activity; and iii) consolidation and retrieval of the memory trace from a fearful event. It remains to be determined if the cerebellum is also directly involved in the emotional aspects of defensive behaviour.

In relation to motor control the cerebellum receives information from numerous ascending and descending pathways which are relayed either through the mossy fibre system or the climbing fibre system. Central to all major theories of cerebellar operation is the dynamic interplay between these two cerebellar inputs. Mossy fibres are thought to provide a moment-by-moment update to the cerebellum of an evolving movement (see next section), while climbing fibres are generally considered to relay error signals and induce synaptic plasticity to adapt on-going movements or learn new behaviours [25]. An important outstanding question is how these two systems combine to regulate different emotional behaviours.

## The role of the cerebellum in motion control

### Fundamental anatomical features of the cerebellum

Several pioneering works have established unequivocally that the cerebellum is a major structure in the CNS for the control of voluntary motion (Table 1). Three main fundamental anatomical features have emerged. First, cerebellar cortex is organized into a myriad of functional units called microzones [26]. A microzone is defined as a group in the order of 1000 Purkinje cells (PCs) all having the same somatotopic receptive field. These PCs are arranged in a long, narrow strip, oriented perpendicular to the cortical folds. Second, the cerebellum is organized in multiple modules. A module is a conglomerate of several non-adjacent parasagittal bands of PCs projecting to specific areas of cerebellar nuclei (CN) and gating segregated projections from the inferior olive [27]. Third, there are segregated loops between the cerebellum and prefrontal cortex, parietal cortex, paralimbic cortex and superior temporal sulcus. The anatomical circuits that link the cerebellum with the cerebral cortex are arranged into a two-stage feed-forward loop and a two-stage feedback loop comprising multiple parallel and partially overlapping subcircuits [28]. Both motor corticopontine projections and association cortex projections (from prefrontal, posterior parietal, superior temporal polymodal, parastriate, posterior parahippocampal and cingulate regions) are somatotopically organized in the pons. From the somatotopical standpoint, the cerebellum contains one representation in the anterior lobe (lobules I-V) and a second one in lobule VIII. The anterior lobe, adjacent parts of lobule VI and lobule VIII receive spinal afferents via the spinocerebellar tracts and are reciprocally linked with the medial and dorsal accessory nuclei of the inferior olivary complex, which receive spinal afferents [29, 30]. By contrast to these anatomical arrangements, the lateral portions of the human cerebellum (remainder of lobule VI, lobule VII: vermal lobule VIIa, Crus I, Crus II, lobule HVIIB) have no connections with the sensorimotor areas of cerebral cortex and no spinal input [30]. These areas are reciprocally linked with the association areas of the cerebral cortex concerned with higher order behaviour, via the dentate nucleus which has expanded through evolution along with the posterior lobe of the cerebellum and the cerebral cortical association areas [28, 31]. This dichotomy between the sensorimotor cerebellum and the lateral portions of the cerebellum (cognitive cerebellum) is one of the anatomical substrates to explain the subdivision of the cerebellar syndrome into 3 clinical syndromes: the vestibulocerebellar syndrome, the cerebellar motor syndrome and the cerebellar cognitive affective syndrome/Schmahmann’s syndrome [32, 33].

**Table 1:** Scientists who made a major contribution to cerebellar research^a^

Family Name	Year	Discovery
Malacarne	1776	Cerebellar foliation
Rolando	1809	Cerebellar lesions impair posture and voluntary movement
Flourens	1824	Cerebellar lesions are linked to muscle incoordination
Magendie	1824	Cerebellar peduncles contribute to coordination
Luciani	1891	triad of atonia/asthenia/astasia
Lugaro	1894	Description of the elements of the cerebellar cortex
Babinski	1899-1906	Description of asynergia and adiadochokinesia
Sherrington	1900	Cerebellum regulates a complex proprioceptive system
Holmes	1904-1939	Detailed reports on cerebellar dysmetriadow and kinetic tremor
Cajal	1911	Description of the fine network structure of the cerebellar cortex
Larsell	1937-1972	Identification of the 10 cerebellar lobules (I-X)
Eccles	1963-1967	Studies on neuronal connectivity in the cerebellar cortex
Voogd	1964-1969	Longitudinal organization of the cerebellum
Ito	1964	Purkinje neurons inhibit cerebellar nuclei neurons
Gilman	1969	Cerebellum tunes the activity of muscle spindles
Marr and Albus	1969-1971	Computational theory of learning
Llinas	1974	Strong electrotonic coupling between inferior olivary cells
Hallett	1975	Cerebellum controls the timing of muscles discharges
Oscarsson	1976	Microzone as the functional unit of the cerebellar cortex
Gilbert and Thach	1977	Motor adaptation is associated with an increase in the firing of complex spikes
Ito and Kano	1982-1984	Long-term depression (LTD) Cerebellar microcomplexes
Haines and Dietrichs	1984	Reciprocal anatomical connections between the hypothalamus and the cerebellum
Mugnaini	1994	Discovery of unipolar brush cells
Schmahmann	1998	Description of Schmahmann’s syndrome
Strick	2005	Reciprocal anatomical connections between cerebellum and basal ganglia

^a^Modified from Manto and Haines, 2012

Studies on resting-state functional connectivity (fcMRI) have demonstrated a key-contribution of the cerebellum in the intrinsic connectivity networks (ICNs) which overlap to a large degree with maps of structural connectivity derived from diffusion tensor imaging (DTI) tractography [34–36]. fcMRI is growingly used to assess the contribution of cerebellum in cognitive and 1affective operations [37–41].

It was assumed that cerebello-cortical loops and cortico-basal ganglia loops interacted only at the cerebral cortex level. Transsynaptic tracer studies have revealed (a) a disynaptic pathway linking the striatum and dentate nucleus via the thalamus, and (b) a disynaptic pathway linking the cerebellar cortex and the subthalamic nucleus (STN) via pontine nuclei [42]. The STN is divided into three anatomico-functional compartments (sensorimotor, associative and limbic) with distinctive neuronal firing patterns [43]. Projections from STN to the cerebellum originate from the three divisions.

### Fundamental neurophysiological features

The discovery by Ito that inhibitory post-synaptic potentials (IPSPs) are induced monosynaptically in neurones of Deiters’ nucleus by stimulation of the cerebellar cortex at the anterior vermian part was a breakthrough in cerebellar research [44]. This was the first definite evidence that PCs exert an inhibitory effect upon CN. PCs discharge either with simple spikes (high rates: 30 to 100 Hz; driven by mossy fibres inputs to granule cells) driven by parallel fibres input, or with complex spikes (low rates: about 1 Hz; driven in part by the large voltage-gated calcium conductance in the dendrites of PCs) as a result of climbing fibre activation producing a distinctive high-frequency burst of spikes [45]. Simple spikes modulate weakly during passive movements, but strongly during active movements [46]. PCs receive a massive convergence of inputs from parallel fibres, a factor which allows an integration of neural information from distinct sources. The activities of complex spikes following discharges in the inferior olivary complex are viewed as a teaching signal (trial-and-error during a learning process) or a motor clock signal which provides a rhythmic neural signal used to time motor processes for time perception to production of timed movements [47–49]. Others have suggested that complex spikes exert a synchronization effect in the cerebellar cortex, both within and between the cerebellar microcomplexes [50].

A very robust property of cerebellar nuclear neurons recorded in brain slices is their ability to fire rebound spike bursts following strong hyperpolarization induced by current injection [51]. These rebounds are both seen in GABAergic and glutamatergic cerebellar nuclear neurons. This is a mechanism by which PC inhibition is tuned in CN output spiking. In other words, a real sculpting of patterns of discharges of CN occurs as a combination of inhibition from cerebellar cortex and rebound spiking.

### General rules of motor control

Optimal strategies are required to perform motion with accuracy, given the highly complex non-linear biomechanical features of the human body, including the muscles and joints, and the numerous interactions with the environment. The CNS copes with noise and delays, which are inherent to biology and also motion. The notion of noise in biological signals includes both the input noise and the internal noise. Noise may also fluctuate with time or according to a particular sensori-motor context. Noise may corrupt a signal of interest [52]. Therefore, dealing with noise is of great importance for adequate motor control. A high degree of adaptability and modifiability in the operational mechanisms underlying motor control is required, especially for learning procedures. Cerebellum is a structure characterized by a high degree of plasticity and faces the issue of noise given its numerous afferent connections and the multiple cerebello-cerebral loops running in parallel. Time delays are related to feedback control mechanisms [53].

### Purkinje cells (PCs), synaptic plasticity and the theory of internal models

According to David Marr [54]: the simple and repeated fine structure in the cerebellum is ideal for motor learning. Fritz Kahn made an analogy to radishes (PCs) in bread slices (cerebellar folia) [55]. This is more than a historical curiosity because this analogy helps understanding the importance of parallel fibers (PFs). Indeed, cerebellum is composed of a redundant architecture, with PFs running through the molecular layer parallel to the long axes of the cortical folds known as folia and thus linking the PCs [56]. The PFs imply a delay line which is dependent on the length of the fibre.

Adjusting future movements implies the implementation of predictive mechanisms. This is essential for planning procedures. The current prevailing theory assumes that the cerebellum anticipates the consequences of actions driven by the cerebral cortex. Internal models provide for representations of the input–output properties of the motor apparatus [57, 58]. They are grouped in 2 general classes: forward models use the commands for an action and information about the present state to predict the consequences of the action. Forward model requires two inputs: (a) an efference copy (which corresponds to a copy of a given motor command) from a controller and (b) an afferent sensory signal sensing the actual state of the motor system. Inverse models transform a desired outcome or effector state into the necessary commands to achieve that state. In terms of flow of information, the inverse model can be considered as the inversion of the forward model. There is growing evidence that the cerebellum acquires and stores internal models. For instance, PCs simple spike firing during arm movements have several properties consistent with a forward internal model [57]. PCs in lobules IV–VI of the intermediate and lateral cerebellum carry signals related to the position, the direction, the amplitude and the speed of movement.

### Cerebellar dysmetria

The terminology of ataxia of limbs encompasses dysmetria (hypermetria: overshoot, hypometria: undershoot), dysdiadochokinesia, cerebellar tremor (action tremor, postural tremor, kinetic tremor), isometrataxia, disorders of muscle tone (mainly hypotonia observed especially in severe acute lesions of the cerebellum in children) and impaired check and rebound [59].

Limb dysmetria is as an error in trajectory due to an abnormal range, rate and/or force of motion [60, 61]. In monkeys and in humans, cerebellar dysmetria is speed-sensitive, is sensitive to inertia, shows an increased inter-trial variability, is characterized by an increased curvature of movement, and shows an impaired initiation of movement [32]. Dysmetria is most often composed of hypermetria during fast movements. Increasing inertia worsens hypermetria and addition of inertia to the moving limb transforms a normometric movement into a hypermetria [62, 63]. Cerebellar cooling is associated with decreased phasic discharges in neurons of the motor cortex and decreased magnitude in the agonist muscle, supporting the notion that the cerebellum contributes to the genesis of agonist commands during voluntary movements [64]. The excitability of the motor cortex contralaterally to a cerebellar lesion involving CN is decreased [65]. Cerebellar dysmetria can be explained by biased internal models of limb dynamics [66]. Underestimation of inertia causes an overshoot and overestimation of inertia causes an undershoot. The impaired check and rebound might be related to errors in the adaptation to unanticipated external perturbations.

### Prediction of sequences

**Fig. 1:**
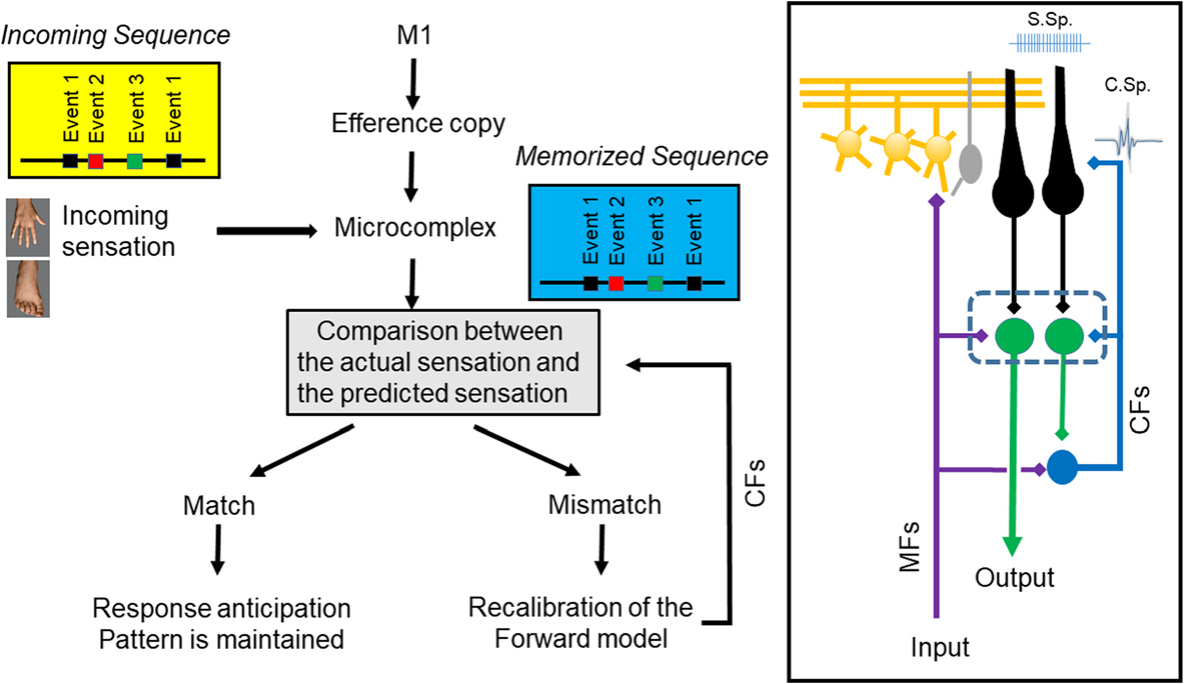
Cerebellum detects similarities and discordances between predicted and actual sequences of events. The incoming sensation activate a microcomplex especially via activation of mossy fibres (MFs). Selected bands of Purkinje neurons are activated, resulting in a sculpting of patterns in the cerebellar nuclei. In case of discrepancy between actual signals and expected sequence, the forward model is recalibrated via activation of the climbing fibres (CFs) of the olivo-cerebellar tract. Right panel: schematic representation of the cerebellar circuitry. Parallel fibres emerging from granule cells (in orange) generate simple spikes (S. Sp.) at the level of Purkinje neurons (in black). Purkinje neurons target cerebellar nuclei neurons (in green). The activation of the inferior olivary complex (in blue) triggers a complex spike in the cerebellar cortex

The cerebellum is a site of several forms of plasticity involved in adaptation and is a master-piece for predictions at the motor and cognitive level, including working memory and attention [67]. The key-functions of adaptation and estimation of the mechanical state of the limbs have been approached by pioneers of cerebellar research [68]. An example of how the cerebellum handles predictions for locomotion has been provided by Molinari (Fig. 1). The monitoring of events and the comparison with stored sequences occur within a cerebellar microcomplex [69]. When a match is detected, a given pattern is expected and a prediction is built. In case of mismatch, an error signal is generated within the olivo-cerebellar system and the prediction system is corrected. In case of discrepancy, the activity of the cerebellum increases and a large area of the cerebral cortex is alerted with enhancement of neuronal excitability [70]. This is fully compatible with a role of the cerebellum as a predictor, not only in the sensori-motor domain but also in cognitive/behavioural domains [70, 71].

## Cerebellum and motor timing in movement disorders

Time perception is an essential element of our conscious and subconscious experience, providing synchrony and foundation for reliable interaction with the surrounding environment, both in the retrospective and the prospective domains. Modern theories delineating internal timing networks generally share the view that temporal data is processed in a distributed network with fluctuating involvement of individual components based on the specific task demands [72]. Research efforts have been increasingly directed towards understanding the patterns of neuronal activity and their behavioural correlate, implicating several regions including cerebellum, basal ganglia, frontal cortex and posterior parietal cortex [73] in both healthy individuals and specific patient populations. The notion of correct cerebellar function being crucial for successful analysis of temporal information is definitely not new [74] and it has gained momentum in the recent years, being underscored in neuroimaging [75, 76], neurophysiological [77, 78] and in clinical studies of patients suffering from varying degree of cerebellar pathology, ranging from predominantly cerebellar defects with deleterious impact on the quality of life [79, 80] to categories with plausible, even though often disputed affection of the cerebellum [81].

It has been more than 10 years since the landmark study on predictive motor timing, which hypothesized the crucial role of the cerebellum in integrating incoming visual information and motor outputs to predict the future state of the surroundings based on the findings in patients suffering from spinocerebellar ataxia [79]. A simple button press releasing a projectile to hit a moving target proved too much of an adversary for them and in the following fMRI project using the very same task, their decreased performance was confirmed to be related to the reduced cerebellar activation [80]. The quantitative character of the difference in activation raised an intriguing notion that, contrary to a lesion, atrophy does not lead to qualitative reorganization of processing at the level of cerebellum, even though compensatory engagement of higher order brain networks has been shown in Friedreich ataxia [82]. These findings accord well with the previous report of defective adaptation of anticipatory muscle activity to the changes in the environment in the patients with cerebellar disorders [83].

In an extension of the spectrum of clinical units in this research framework, essential tremor patients have failed to reach the same cerebellar activation level as the controls in a simple motor timing task as finger tapping [84]. Moreover, patients with essential tremor affecting also the head exhibited fundamental problems with predictive motor timing, too, whereas Parkinson’s disease (PD) patients seemingly performed at the same level as controls [85, 86]. Indeed, simple fMRI activation analysis confirmed affection of both the cerebellum and the basal ganglia in PD patients [81], but the true nature of this finding, exposed only in the subsequent analysis of effective connectivity, lies in profound cortical reorganization of the brain affected by PD, struggling to maintain the original performance [87].

Even cervical dystonia (CD), a disease traditionally conceptualized as a basal ganglia disorder, has lately also seen a mild turn in the hypothesized pathophysiological origin to a network, which also comprises of other parts of the brain, including the cerebellum [88]. Interestingly, when facing the very same need for predictive timing in the sub-second spectrum, CD patients reach lower success rates than the normal population [89]. The following complex neuroimaging study searching for the basis of this finding implicated a network of affected brain structures and, more importantly, unveiled an extensive communication defect between the cerebellum and the basal ganglia in the connectivity analysis [90]. The picture emerging here provides a sound rationale for considering even cerebellum as a possible future target for dystonia treatment [91, 92].

Nonetheless, the extent of disruption of cerebellar processing capacities remains notably underexplored in many common movement disorders. Despite the prevalence of these diseases and their substantial impact on the quality of life and healthcare costs, scientific progress on the path to a real cure of neurodegenerative disorders is far from sufficient. And it is only by looking on the entire complex of clinical deficits and all the pathological alterations to the neural networks, that we may come to reach for a true cure.

## The role of the cerebellum in tremor genesis – current concept of cerebellar tremor

The cerebellum is known to be involved in all tremor syndromes, being part of a complex network responsible for tremor genesis. According to Elble, it is not surprising as the cerebellum is “exquisitely sensitive to all deviations of the intended movement” [93].

The cerebellum plays a role in essential tremor and tremor in Parkinson’s disease which are both well studied, although their pathological mechanisms are still not fully elucidated [94].

Cerebellar tremor is a symptomatic tremor associated with cerebellar injuries. Although, it is a well-established clinical evidence that cerebellar lesions may cause tremor, as it has been stated in a recent consensus paper on cerebellar functions, the nature and genesis of this tremor type has not been resolved [32]. In order to understand the role of the cerebellum in the whole tremor network, we have to focus on such conditions which affect exclusively the cerebellum or its input and output connections.

### Pathophysiology of cerebellar tremor

In 1893, Ferrier and Turner experimentally lesioned different cerebellar structures in 26 monkeys and postoperatively observed action tremor together with a set of other behavioural changes [95]. This investigation suggested that focal cerebellar lesions might induce tremor. In the 1970’s Larochelle et al., and Poirier et al. lesioned the dentate nuclei (DN) and the superior cerebellar peduncle (SCP) but did not find tremor. Tremor could be detected only after injection of harmaline, which duplicates the effects of monoaminergic disturbances [96, 97]. It was demonstrated that cooling of the DN can induce impaired coordination of arm movements and tremor [98]. Flament and Hore showed that tremor characteristics were different in isotonic and isometric conditions after cooling the DN of animals. This experiment raised the suspicion that multiple mechanisms underlie cerebellar tremor: activity of a central oscillator could be modulated by peripheral mechanisms as tremor characteristics changed after proprioceptive feedback from the limb was altered [99].

The first observations in humans were made by Gordon Holmes in 1922. He identified three different types of cerebellar tremor and noted that the underlying pathophysiology was different. However, according to his concept all cerebellar disturbances were due to muscle tone impairment and muscular asthenia. He also noted that at the beginning of the task (holding a posture) the affected limb could even be more stable than the unaffected one as the fine vibratory physiologic tremor was often absent in the affected limb [60].

Nowadays, there is a consensus that cerebellar tremor develops when the dentato-rubro-thalamic system (DRT) is affected [93]. According to a DTI analysis, cerebellar tremor might develop even without cerebellar lesion, if the DRT is damaged [100]. According to this concept, cerebellar tremor is generated as a dysfunction of a network rather than that of the cerebellum itself. This is an important finding, however it does not explain the origin of cerebellar tremor as the DRT is involved in all tremor syndromes. The involvement of the DRT in all tremor syndromes makes it an ideal deep brain stimulation (DBS)-target for tremor treatment [101].

### Imaging in cerebellar lesion related to tremor

As stated in the recent consensus paper on signs and symptoms in the cerebellar syndrome, it is not clear why some patients with cerebellar lesions exhibit tremors, and others do not [31]. Current techniques of neuroimaging provide new possibilities for more precise correlation between lesion anatomy and functional loss [102]. Functional MRI studies have provided new somatotopic maps of the cerebellum. It has been demonstrated that arm and finger movements are represented both in the anterior [103] and posterior lobes [104]. DTI analysis of patients with cerebellar lesions might provide new insight into white matter loss and recovery in the setting of cerebellar tremor. New atlases have been developed, showing 7precisely the cerebellar lobules [105, 106], deep nuclei [107] and white matter [108]. These imaging aids and methods make it possible to overcome inter-subject and inter-study variability. More precise anatomical correlations might improve our understanding of the pathophysiology of cerebellar tremor.

### Clinical presentation and types of cerebellar tremor

According to Louis, nowadays “cerebellar tremor is equated exclusively with intention tremor” in an “over-simplified manner” [32], although Gordon Holmes had already described three types of cerebellar tremor: static tremor (postural), kinetic tremor (the limb is held before the target, it does not reach the target) and tremor in lesions of the SCP which involve mostly the head and trunk [60].

There is consensus that resting tremor does not emerge in cerebellar lesions [109]. The “esting cerebellar tremor” described by Nakamura et al. most probably belongs to the family of Holmes tremor [110].

Late-onset tremors, like palatal tremor which develop months-years after the lesion, have also been described and are most probably due to secondary reorganisation of the involved pathways. Brown at el. described three cases with cerebellar axial postural tremor, which involves the head and the trunk and it is present at rest and in posture, but disappears during voluntary movement [111]. Louis et al. published five cases with “delayed-onset cerebellar syndrome” including intention tremor, which developed 3 weeks to 2 years after stroke or traumatic head injury [112].

Tremor due to cerebellar lesion usually develops ipsilaterally to the side of the lesion. However, patients of Cole et al. presented with bilateral finger tremor even if only one cerebellar hemisphere was affected [113].

### Objective parameters of cerebellar tremor

Animal experiments showed that cooling of the DN resulted in a tremor of 3–5 Hz [98, 99]. Larochelle et al. showed that tremor frequency in isometric contractions decreased to 1.48-2.75 Hz [5]. Frequency of cerebellar tremor in humans was seldom measured: 5-7 Hz was reported by Cole et al. [113], 8-12 Hz was described by Milanov [114]. In one study on patients with delayed-onset cerebellar syndrome, tremor frequency was 1.5-4 Hz [112]. In the Consensus Statement on Tremor in 1998 [109], and in 2017 [115] it is stated that the frequency of cerebellar tremor “is below 5 Hz” (without reference given).

The amplitude of cerebellar tremor is usually low [113] making it hard to notice or distinguish from other low amplitude tremors [114]. To reliably detect tremor caused by cerebellar lesion quantitative recordings should be encouraged.

### Recovery of cerebellar signs after acute cerebellar lesions

The improvement of cerebellar symptoms is dependent on the aetiology of the lesion. For instance in inherited neurodegenerative disorders involving the spinocerebellar pathways the cerebellar symptoms progressively deteriorate. The first objective study which investigated the time course of recovery of cerebellar symptoms after acute cerebellar stroke, quantified the degree of ataxia/bradykinesia. This study showed that most of the improvements occurred in the first 2 weeks after the acute lesion, and in some patients amelioration continued until the third month [116]. Although it is a clinical evidence that cerebellar tremor caused by acute cerebellar lesions regress in a relatively short time, to date there is no objective study on the recovery of cerebellar tremor after acute cerebellar damage.

### The link between essential tremor and the cerebellum and the need for further studies

Neuroimaging studies [117, 118] and post-mortem studies [119, 120] demonstrated that the cerebellum is a key region in the pathogenesis of essential tremor. However, the exact role of the cerebellum in essential tremor-related mechanisms has not been elucidated [121]. The functioning cerebellum seems to be necessary for essential tremor. It has been reported that after vascular lesion of the cerebellum, tremor disappeared on the ipsilateral side in an essential tremor patient [122]. Contrary to this, cerebellar hemispherectomy triggered essential tremor in a patient suggesting the role of the thalamus in tremor genesis in essential tremor [123]. It is an established fact that essential tremor patients exhibit cerebellar signs [124, 125], and tremor in these patients steadily progresses and never recover. This underlines the important difference between neural plasticity processes in neurodegenerative disorders and acute cerebellar lesions.

Our current knowledge on cerebellar tremor is mostly based on animal experiments. Human studies are scarce and have provided insufficient results so far. Diversity of the clinical presentation of tremors caused by cerebellar lesions is not recognized. New imaging techniques applied in patients with well characterised lesions might bring deeper understanding of the mechanism of cerebellar tremor. Human studies determining the quantitative characteristics of tremor induced by cerebellar lesions are also much needed.

## General conclusion

We have underlined multiple facets of cerebellar functions. Despite its highly homogeneous and cristalline anatomical structure, we still lack a consensus on the operational mode of the cerebellum, one of the top mysteries for CNS disorders. However, no doubt that progress has been made in the theories of cerebellar functions. The universal cerebellar transform embedded within the dysmetria of thought theory is an example of the effort to unify the operational mode of the cerebellum [126].

Its dense connectivity with cerebral cortex, thalamic nuclei, brainstem nuclei and spinal cord, as well as its critically high number of neurons put the cerebellum in a unique position for a participation in cognitive, affective and sensorimotor operations. This special session has highlighted this aspect by taking fear behaviour, motor control, timing contributions and tremor as 4 examples of productive fields of research.

## Abbreviations

DRT: Dentate-rubro-thalamic system
DTI: Diffusion tensor imaging
IPSPs: Inhibitory post-synaptic potentials
PAG: Periaqueductal grey
STN: Subthalamic nucleus
TTX: Tetrodotoxin
